# Tumor reactivity assessment using clonal expression reveals tumor reactive CD8^+^ T cell heterogeneity across solid tumors

**DOI:** 10.3389/fimmu.2026.1815974

**Published:** 2026-04-16

**Authors:** David Monteiro, Jack Denebeim, Anne E. Dodson, Ashish Yeri, Mitali Ghose, Meghan Travers, Sophie Capobianco, Conor Calnan, Gustavo J. Martinez, Charles H. Yoon, Karrie Wong, Micah J. Benson, Dipen Sangurdekar

**Affiliations:** 1 KSQ Therapeutics, Inc., Lexington, MA, United States; 2 Brigham and Women's Hospital, Boston, MA, United States; 3 Harvard Medical School, Boston, MA, United States

**Keywords:** tumor-infiltrating lymphocytes (TIL), tumor-reactive T cells (TRT), machine learning, tumor microenvironment (TME), cancer immunotherapy, computational immunology, AI-driven biomarker discovery, multi-modal single-cell analysis

## Abstract

**Introduction:**

Tumor infiltrating lymphocytes (TIL) drive the anti-tumor activity of a broad class of immunotherapies. *In situ* TIL are composed of T cells that recognize tumor antigens (Tumor Reactive T cells, or TRTs) as well as bystander T cells with specificity for other antigens. TRT clonotypes are associated with a unique and tumor-driven exhausted transcriptional state, enabling single-cell RNA sequencing (scRNA-seq)-based predictive models for TRTs using experimentally validated clone labels.

**Methods:**

In this study, a clonotype-level CD8^+^ TRT classifier (TRACE) was built using an aggregated dataset of validated tumor reactive clonotypes and associated scRNA-seq data from multiple publications that overcomes the limitations of training on a single dataset, donor, or indication. TRACE does not require dataset manipulation for training or prediction, enabling it to be easily applied to new test datasets as they emerge.

**Results:**

TRACE exhibited robust performance on held-out TIL and PBMC clones - achieving a mean Matthews correlation coefficient of 0.84 and F1-score of 0.85 - comparable to or outperforming other TRT prediction methods. We experimentally confirmed the reactivity of TRACE-identified TRT clones by co-culturing *ex vivo* expanded TIL with an autologous melanoma tumor cell line. Finally, we applied TRACE to evaluate the frequency of TRTs across hundreds of patient samples from multiple tumor atlases spanning lung, colorectal, and pancreatic cancer. TRACE scores were observed to be significantly higher in exhausted CD8^+^ T cells in tumors but not in exhausted cells in normal adjacent or non-cancer samples, suggesting specificity towards identifying tumor-antigen experienced T cells.

**Conclusion:**

TRACE is a tumor reactivity scoring algorithm released with open model weights that can be applied to tissue or blood single-cell RNAseq datasets. Its application should be of general interest for characterizing the fraction of TRTs in TIL and for establishing correlations with clinical response to immunotherapies.

## 1 Introduction

Immune checkpoint blockade (ICB) and adoptive cell therapies (ACT), including tumor infiltrating lymphocyte (TIL) therapy, have demonstrated durable clinical benefit across multiple cancers, particularly immunologically hot tumors such as melanoma (1, 2). A consistent biological determinant of response to these immunotherapies is the presence of cytotoxic CD8^+^ T cells within the tumor microenvironment (TME), where they mediate direct tumor cell killing and shape local immune dynamics (1, 3–5). However, the mere abundance of intratumoral CD8^+^ T cells is insufficient to explain therapeutic efficacy, as tumors often harbor a heterogeneous mixture of tumor reactive and bystander T cells that differ markedly in antigen specificity, transcriptional state, and functional potential (6). Recent studies have established that a substantial fraction of CD8^+^ T cells infiltrating tumors are bystander T cells, recruited independently of tumor antigen recognition and lacking direct anti-tumor activity (6–8). In contrast, increased infiltration of tumor reactive T cells (TRTs) - defined by recognition of tumor-derived antigens, including neoantigens - is preferentially associated with favorable prognosis and response to immunotherapy (9, 10). In melanoma and other inflamed tumors, therapeutic response correlates more strongly with the abundance, clonal dynamics, and functional state of TRTs than with total CD8^+^ T cell density (6, 8, 11–14). These findings underscore the importance of discriminating tumor reactive from non-tumor reactive T cells within complex intratumoral immune populations.

Antigen-experienced TRTs occupy a distinct transcriptional niche within the TME. Multiple single-cell and functional studies have shown that TRTs frequently adopt a tissue-resident memory-like phenotype characterized by expression of markers such as *ENTPD1* (CD39), *ITGAE* (CD103), and chemokines including *CXCL13*, alongside features of chronic antigen stimulation and exhaustion (3, 8, 10, 11, 15). Importantly, these transcriptional programs are enriched for tumor reactivity across cancer types and have been linked to both response to checkpoint blockade and persistence following ACT (3, 11, 16). Nevertheless, no single marker or gene signature fully captures tumor reactivity, and substantial overlap exists between TRTs and non-reactive exhausted or activated T cell states.

This limitation has direct implications for ACT. Clinical and translational studies demonstrate that enrichment of tumor reactive T cells within TIL products using neoantigen screening or phenotypic selection strategies can improve therapeutic efficacy (8, 17–21). However, current approaches to identifying TRTs rely on labor-intensive functional assays, peptide-MHC multimers, or bulk phenotypic proxies that are difficult to scale and are often incompatible with routine single-cell profiling. Computational approaches applied to single-cell transcriptomic data offer a principled opportunity to address this gap. Recent single-cell studies have substantially advanced the transcriptional characterization of TRTs by integrating scRNA-seq with TCR sequencing and functional validation, revealing conserved programs associated with chronic antigen stimulation, tissue residency, and exhaustion-like states (22–26). Building on these datasets, several computational approaches have attempted to infer tumor reactivity directly from transcriptomic profiles, including marker-based scores, gene signatures, and supervised machine-learning classifiers trained on experimentally validated clonotypes. Recent machine-learning approaches such as TRTpred (26), MANAscore (27), NeoTCR8 (24), and predicTCR (28) represent important steps toward computational identification of tumor reactive T cells by leveraging experimentally annotated single-cell datasets and TCR-linked supervision.

Despite their advances, existing methods share important limitations. First, most models are trained on small, experiment-specific datasets derived from individual studies or tumor types, limiting statistical power and robustness to cohort-specific effects. Second, training data are typically not augmented across experiments or platforms, and model weights are not shared, hindering reproducibility and reuse as new datasets emerge. Third, many approaches require extensive single-cell normalization, batch correction, and hand-tuned feature selection, introducing dataset-specific assumptions and constraining generalizability across sequencing depth, chemistry, and platform. Importantly, while multiple studies demonstrate that cells sharing an identical TCR clonotype can occupy diverse transcriptional states within the TME (3, 11, 29–31), existing methods (except TRTpred and predicTCR) largely operate at the single-cell level and do not explicitly model intra-clonal heterogeneity or leverage clone structure during learning. Together, these limitations motivate the development of a more scalable, robust, and clonotype-aware framework for inferring tumor reactivity from single-cell transcriptomic data.

To address these challenges, we introduce TRACE, a machine-learning framework designed to infer tumor reactivity from scRNA-seq while explicitly accounting for clonal structure and technical variability. In the absence of clonal information, TRACE can be applied to individual cells. When TCR information is available, TRACE incorporates a clone-summarizing strategy in which cells sharing a TCR clonotype are aggregated using optimized parameters that preserve biologically meaningful heterogeneity while reducing noise driven by stochastic gene expression. To further stabilize learning across datasets, TRACE employs expression binning, which discretizes transcript abundance into robust ordinal representations that mitigate sensitivity to sequencing depth, platform differences, and batch effects, while dramatically reducing training time. Model training is guided by systematic hyperparameter optimization, enabling consistent performance across heterogeneous cohorts without manual tuning. Finally, TRACE is released with versioned, openly shared model checkpoints, allowing the community to apply, benchmark, and incrementally update the model as new annotated datasets become available. Collectively, these design choices enable TRACE to overcome key limitations of prior approaches and provide a generalizable, extensible foundation for transcriptome-based identification of tumor reactive T cells.

We performed *in silico* benchmarking of TRACE against other methods with publicly available code or gene signatures and found that TRACE delivers comparable or superior performance across holdout datasets spanning multiple cancer indications. We further experimentally validated TRACE in melanoma by identifying tumor reactive clones through T cell activation, as indicated by 4-1BB expression, in a co-culture assay using *ex vivo* expanded TIL and autologous tumor cells. To demonstrate TRACE’s scalability and utility across diverse datasets, we applied the model to multiple single cell tumor atlases and examined its associations with tumor and clinical metadata. Consistent with its design, TRACE scores were enriched in clonally expanded, exhaustion associated T cells within the tumor but not in adjacent normal tissues. Together, these results highlight TRACE as a robust and generalizable tool for identifying and characterizing tumor reactive T cells across heterogeneous single cell datasets spanning multiple cancer indications.

## 2 Results

### 2.1 Optimization of TRACE model parameters and training strategy

To build TRACE, we first compiled six scRNA/scTCR-seq datasets spanning multiple indications containing CD8^+^ TIL transcriptomes with matched, experimentally verified tumor reactivity labels (22–26, 32) (**Table 1**). After dataset cleaning, these yielded 9,488 TRTs from 274 clones for training. In addition to experimentally verified non-TRT labels provided by some groups, we supplemented our dataset with a diverse set of probable true negatives. These included published CD8^+^ T cell clones from PBMC samples from healthy (33, 34) or COVID-19-experienced (35) individuals in addition to TIL clones with known antigens present in VDJdb (36). In total, 40,156 cells across 16,465 clones were used for model training.

**Table 1:** Summary of TIL and PBMC datasets used to train TRACE.

Dataset	Tissue context	Experimentally-validated TRT clones (cells)	Experimentally-validated non-TRT clones (cells)	VDJdb-matched non-TRT clones (cells)	PBMC-derived non-TRT clones (cells)
Caushi et al. (22)	Lung (NSCLC)	25 (1512)	0	1332 (5833)	
Hanada et al. (23)	Lung (NSCLC)	19 (576)	0	516 (986)	
Lowery et al. (24)	Melanoma, breast, GI	45 (378)	0	400 (707)	
Meng et al. (32)	PDAC	61 (626)	52 (1510)	0	
Oliveira et al. (25)	Melanoma	46 (5006)	92 (2427)	10 (164)	
Pétremand et al. (26)	Melanoma	78 (1390)	96 (1172)	12 (158)	
Gao et al. (33)	Healthy PBMC				11730 (13317)
Ogura et al. (35)	Healthy + CoV2-experienced PBMC				819 (3226)
10x Genomics PBMC (34)	Healthy PBMC				1132 (1168)
Totals:		274 (9488)	240 (5109)	2270 (7848)	13681 (17711)

TRACE was trained on nine scRNA/scTCR-seq datasets (6 TIL + 3 PBMC) containing CD8+ T cells with paired TCRαβ chain information. For each TIL dataset, TRT clones were identified experimentally by the original authors. Non-TRT clones in TIL were either identified as non-tumor reactive by the authors or contained a TCR_β_ with a known antigen in VDJdb. All clones in PBMC samples from healthy or CoV2-experienced patients were considered non-tumor reactive.

**Figure 1:**
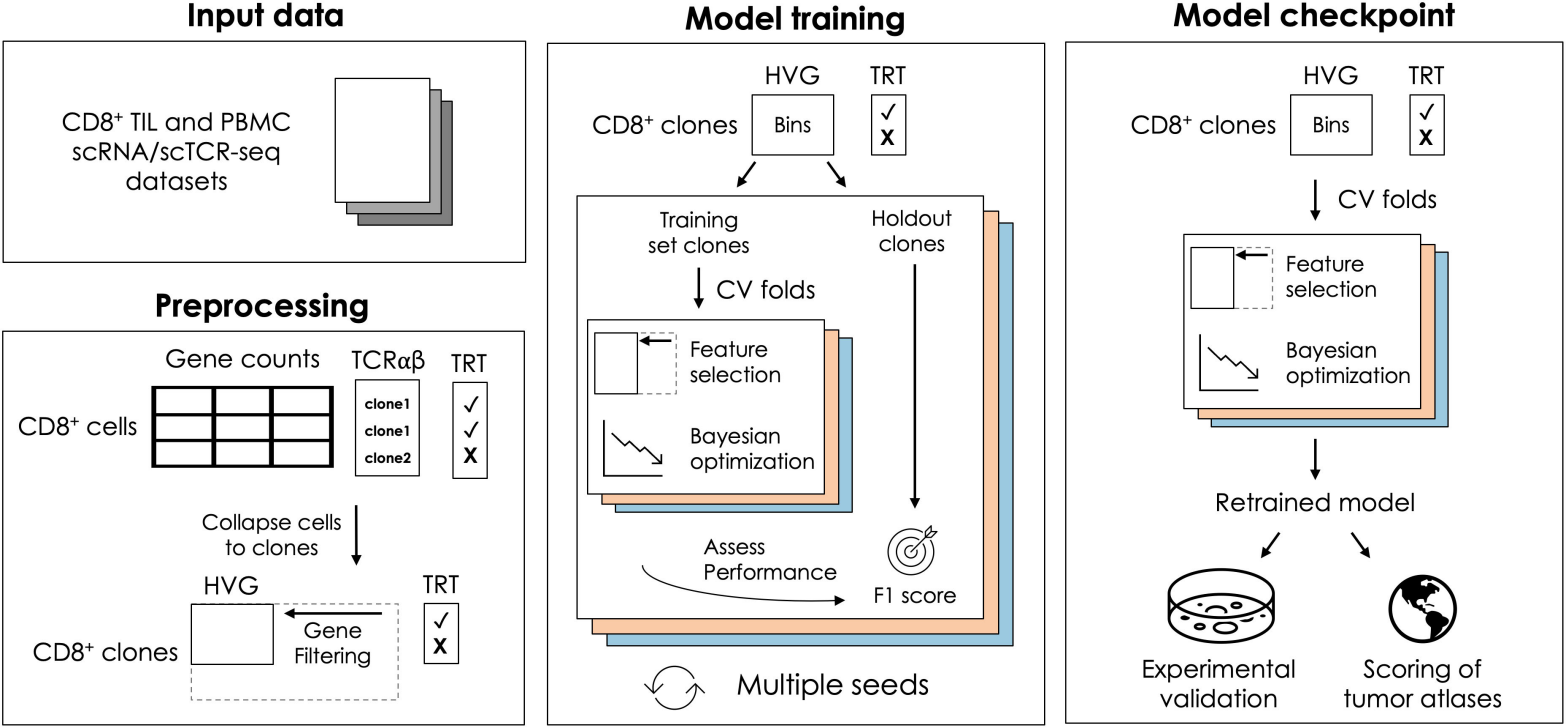
Overview of model training and checkpointing. Single-cell gene expression and paired TCRαβ sequences from TIL and PBMC-derived CD8^+^ T cells were used as inputs for model development. Low-expressing genes were filtered out and gene counts for highly variable genes were collapsed to the clone-level and normalized. To build TRACE, CD8^+^ clones were split into training and holdout sets. Cross-validation folds were used to perform feature selection and Bayesian hyperparameter tuning within the training set, and model performance was evaluated on held-out clones. This procedure was repeated across multiple random train/test splits. A TRACE checkpoint was generated after training on the full dataset using optimized hyperparameters.

To eliminate data leakage between training and test sets, we performed 80:20 train/test splits at the clone-level and rigorously evaluated performance across multiple seeds to select optimal model input parameters (**Figure 1**; Methods; **Supplementary Data**), including 1) the method for collapsing gene expression data to the clone-level, 2) the gene expression transformation method (log normalization or expression binning), and 3) the number and identity of the most important features used in the final model. The final gene list was established by selecting the top *n* ranked genes learned from each training set, defined using median feature importance across all cross-validation folds.

**Figure 2:**
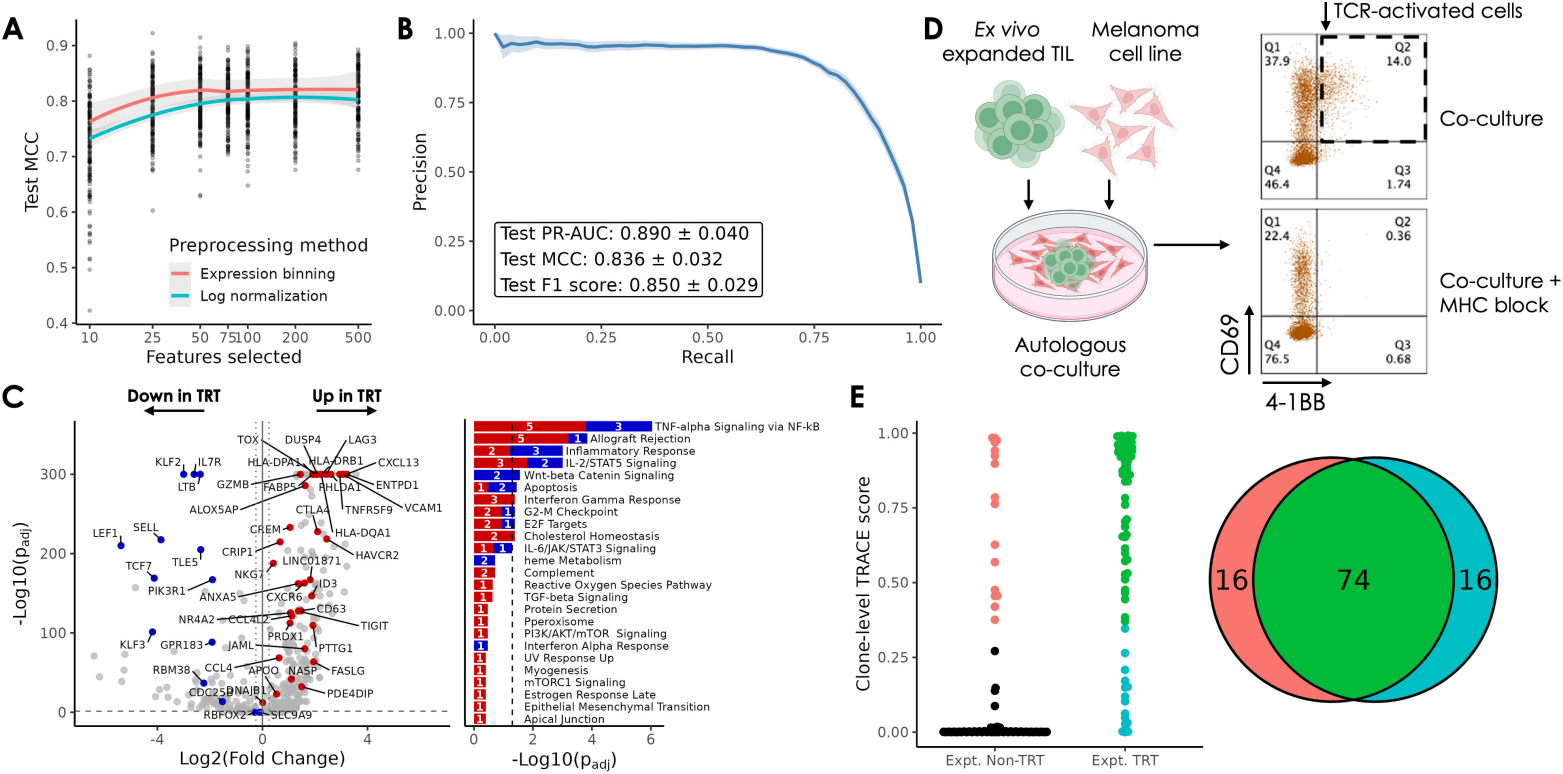
TRACE performance and experimental validation. **(A)** Performance of intermediate TRACE models during model tuning; lines show mean MCC by preprocessing method with a 75^th^ percentile cell-to-clone gene expression collapsing method and Optuna hyperparameter tuning. **(B)** Precision-recall curve (mean with 95% confidence intervals) summarizing TRACE performance across 50 seeds with optimal hyperparameters. Inset shows mean +/- SD. **(C)** Volcano plot highlighting the 50 genes used by TRACE. Blue and red genes are genes used by TRACE that are upregulated in TRT (blue) or downregulated in TRT (red). Grey genes are other highly variable genes used to establish the bins but not used in the final model. Bar plot shows Hallmark pathways containing TRACE genes. On volcano plot, dashed horizontal line represents FDR = 0.05 and dashed vertical lines represent |log_2_(FC)| = 0.25. On bar plot, dashed vertical line represents FDR = 0.05. **(D)** Schematic of autologous co-culture experiments used to validate TRACE performance, generated using BioRender. Flow cytometry dot plots showing CD69⁺4‑1BB⁺ T cells following co-culture in the absence of MHC blockade. **(E)** Summary of overlap between clones predicted to be TRT by TRACE and those exhibiting reactivity during autologous co-culture. Black and red clones are those that did not exhibit reactivity during co-culture either predicted to be TRT (red) or predicted to be non-TRT (black). Green and blue clones are those that exhibited reactivity during co-culture either predicted to be TRT (green) or predicted to be non-TRT (blue). Red, green, and blue clone counts are summarized in the Venn diagram. Flow cytometry dot plots showing CD69^+^4-1BB^+^ T cells following co-culture in the absence of MHC blockade.

Across all combinations of these parameters, we observed that model performance, measured by Matthews Correlation Coefficient (MCC) on the holdout test dataset, increased with the number of retained features up to 50 genes, with only a modest increase at higher model sizes (**Figure 2A**, **Supplementary Figure 1**). Similar tests identified the optimal method for cell-to-clone gene summarization as the 75^th^ percentile of expression levels across cells within a clone, which matched or outperformed the 50^th^ and 100^th^ percentiles (**Supplementary Figure 1**). Likewise, we found that expression binning resulted in intermediate models with higher performance across all conditions tested. A comparison of 3 modeling frameworks - XGBoost, AdaBoost, and Random Forest - identified XGBoost as the most performant on this TRT classification task (**Supplementary Figure 2**).

### 2.2 TRACE accurately identifies validated tumor reactive clones across solid tumors

Using these optimized input parameters (expression binning, 75^th^ percentile, 50 top genes), we performed nested cross-validation across train/test splits from 50 random seeds. Gene selection, hyperparameter tuning, and model training were performed independently on the training data within each iteration, and the trained model was used to predict the tumor reactivity labels of holdout clones. Across these, TRACE exhibited consistently high performance, achieving a mean MCC of 0.84, mean precision-recall area under the curve (PR-AUC) of 0.89 and mean F1 score of 0.85 (**Figure 2B**). Model features were selected from one representative iteration, and the final model was retrained with new hyperparameter tuning on all available data to create a TRACE model checkpoint. Notably, the list of 50 gene features was stable across multiple iterations.

Of the 50 genes comprising the TRACE feature set, 36 genes have higher expression on average in TRT clones vs. non-TRT clones in the aggregated dataset, while the remaining 14 have lower expression (**Figure 2C**). Overall, the up-regulated genes in TRT clones represent a composite phenotype spanning effector-memory, cytotoxicity (*GZMB*, *NKG7*, *CCL4*), and chronic activation/exhaustion (*DUSP4*, *NR4A2*, *ENTPD1*/CD39, *PDCD1*, *LAG3*, *TOX*, *TIGIT*, *CTLA4*). Down-regulated genes included genes expressed in naïve or stem-like T cells (*LEF1*, *IL7R*, *SELL*, *TCF7*, *KLF2*) reflecting the non-antigen experienced or PBMC clones. Gene set and pathway enrichment analysis revealed that among the most-enriched pathways were the TNF-alpha (NF-kB), IFN-gamma, and IL-2/STAT5 pathways, involved in T cell activation and proliferation. Several genes such as *CXCL13*, *ENTPD1*, *TNFRSF9* (4-1BB), and *IL7R*, feature in other published methods (**Supplementary Table 3**, **Supplementary Figure 3**).

To validate TRACE predictions in an independent dataset, we applied TRACE to a melanoma dataset of 46 tumor-reactive clones published by Tan et al. (28). We inferred TCR clonality by analyzing paired TRAV and TRBV gene usage for 33 of these TRT clones from scRNAseq data (Methods), encompassing 1190 cells between them. Of these 33 unique clones, 32 (97%) were predicted to be TRT by TRACE. The single clone predicted to be non-TRT was among the least reactive TRT clones as determined by the authors (0.91% CD107a^+^).

We established an experimental approach to identify tumor reactive clones in *ex vivo* expanded TIL by co-culturing them with an autologous tumor cell line (Methods). After co-culture, we isolated CD8^+^ T cells for analysis by flow cytometry and confirmed the presence of a population of CD69^+^4-1BB^+^ cells that exhibited MHC-mediated functional reactivity (**Figure 2D**). For one melanoma donor, we performed scRNA/scTCR-seq on T cells from tumor starting material (TIL) and on T cells from four biological co-culture replicates where we found the same reactive population - with high expression of the gene *TNFRSF9* (encoding 4-1BB). We used these cells to assign experimentally reactive TRT (Expt. TRT) or non-TRT labels to 1,805 clones. Independently, we applied TRACE to tumor starting material and found high concordance between TRACE predictions and Expt. TRT labels. Since TRACE depends on sequencing performed on limited tumor material and Expt. TRT is assessed on *ex vivo* expanded T cells, not all clones with TRACE labels can be validated experimentally using this approach. Among the 171 clones found in both sample types, TRACE correctly predicted 82% (74/90) of Expt. TRT clones and 80% (65/81) of Expt. non-TRT clones (**Figure 2E**).

### 2.3 TRACE exhibits comparable or superior performance to other tumor reactivity prediction methods

**Figure 3:**
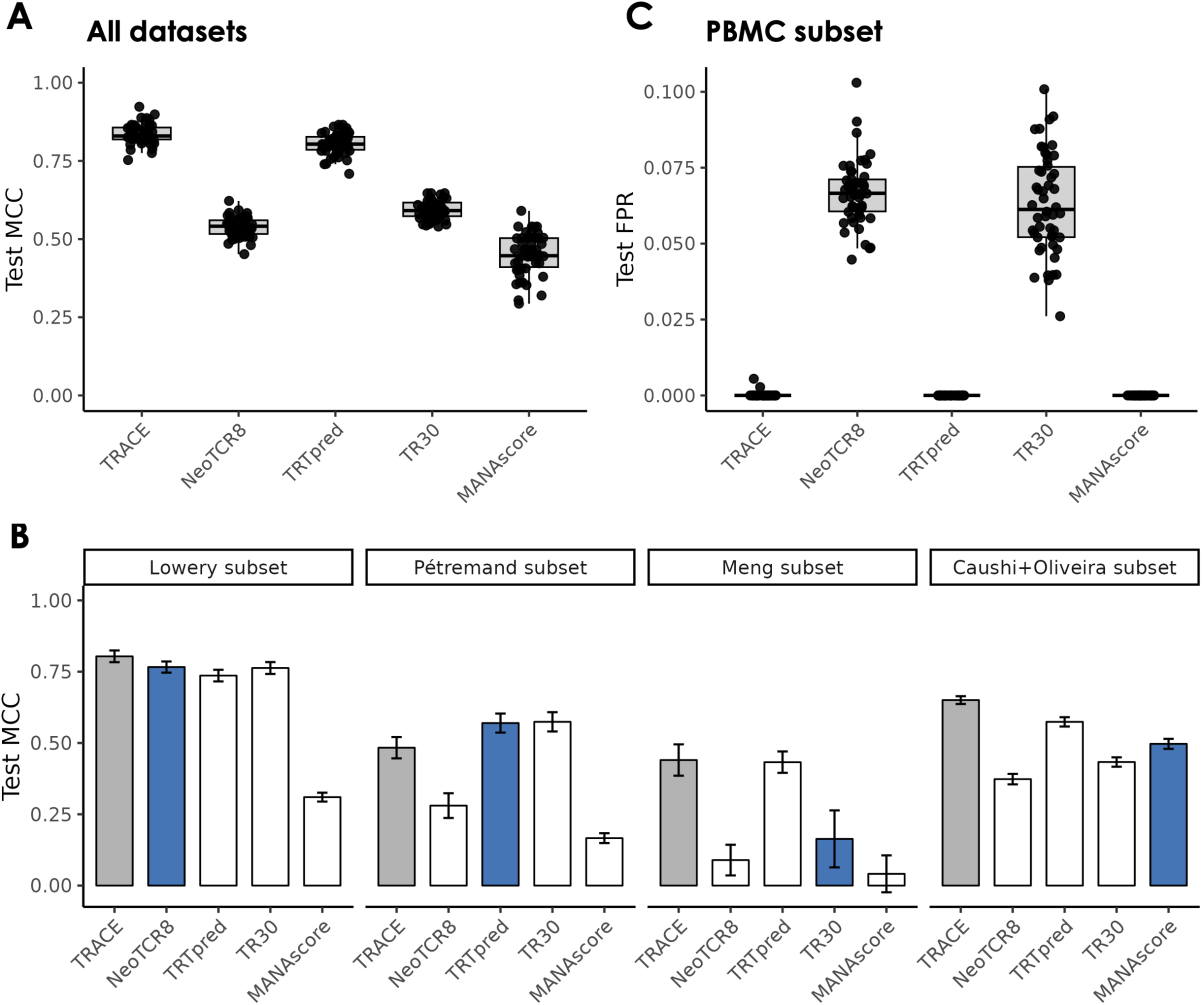
TRACE performance relative to other TRT methods. **(A, B)** Performance (test set MCC) of TRACE and other TRT methods across 50 seeds on test sets containing clones from all available datasets **(A)** and test sets subset for clones from specific datasets **(B)**. Blue bars represent methods trained on the respective datasets. **(C)** False positive rate of all methods across 50 test sets subset for clones from PBMC datasets.

The TRACE model is trained on multiple validated tumor reactivity datasets, which were also used in the corresponding studies to develop tumor reactivity prediction tools. A thorough methodological comparison of these tools is in **Supplementary Table 2**. Since TRACE is trained on an aggregated dataset which was further augmented with negative class labels, we expect the model performance of TRACE to be comparable or superior to all other methods. In addition, TRACE code and model checkpoints are publicly available for re-training and prediction on new datasets, whereas not all TRT methods have either code or checkpoints freely available for thorough benchmarking of TRACE. We implemented and retrained four published TRT methods - NeoTCR8, TRTpred, TR30, and MANAscore - and evaluated them against TRACE on our combined dataset (**Table 2**, Methods). Across the same 50 splits used previously (**Figure 2B**), TRACE models exhibited the highest mean MCC, comparable to TRTpred and outperforming the TR30, NeoTCR8, and MANAscore methods (**Figure 3A**). Subsequently, we investigated the performance of these methods on individual datasets subset from the 50 test splits (**Figure 3B**). Individual methods tended to perform better on the datasets from which they were developed, including NeoTCR8 (trained on data from Lowery et al.) and MANAscore (trained on data from Caushi et al. and Oliveira et al.). TRACE exhibited the strongest performance in 3 of 4 datasets evaluated and had comparable performance across multiple metrics to the TR30 and TRTpred methods on the Pétremand dataset (**Supplementary Table 4**). We also assessed the performance of all methods on a combined PBMC subset, where TRACE exhibited a low false positive rate (FPR) in-line with that achieved by TRTpred and MANAscore (**Figure 3C**). The higher false positive rate of the NeoTCR8 and TR30 methods on this subset could be attributed to the lack of non-TRT-associated genes in these gene sets, which might improve the specificity of TRT classifiers.

**Table 2:** TRT prediction methods implemented and benchmarked against TRACE.

TRT prediction method	Genes (Selection method)	Scoring/predictions	Training data size and tissue context
TRACE	50 genes (model tuning)	XGBoost	40,156 cells (pan-TIL + PBMC’s)
NeoTCR8	243 genes (Seurat FindMarkers)	scGSEA	45,676 cells (Breast, GI, melanoma)
TRTpred	180 genes (edgeR + QLF)	Singscore*	2,845 cells (Melanoma)
TR30	30 genes (Seurat FindMarkers)	UCell	3,365 cells (PDAC)
MANAscore	3 genes (Authors’ selection)	Custom code	253,161 cells (Melanoma, NSCLC + normal lung)

*For TRTpred, the final scoring method was not identified, so Singscore was used.

### 2.4 TRACE precisely identifies antigen-experienced, exhausted CD8^+^ T cells in tumors across multiple indications

**Figure 4:**
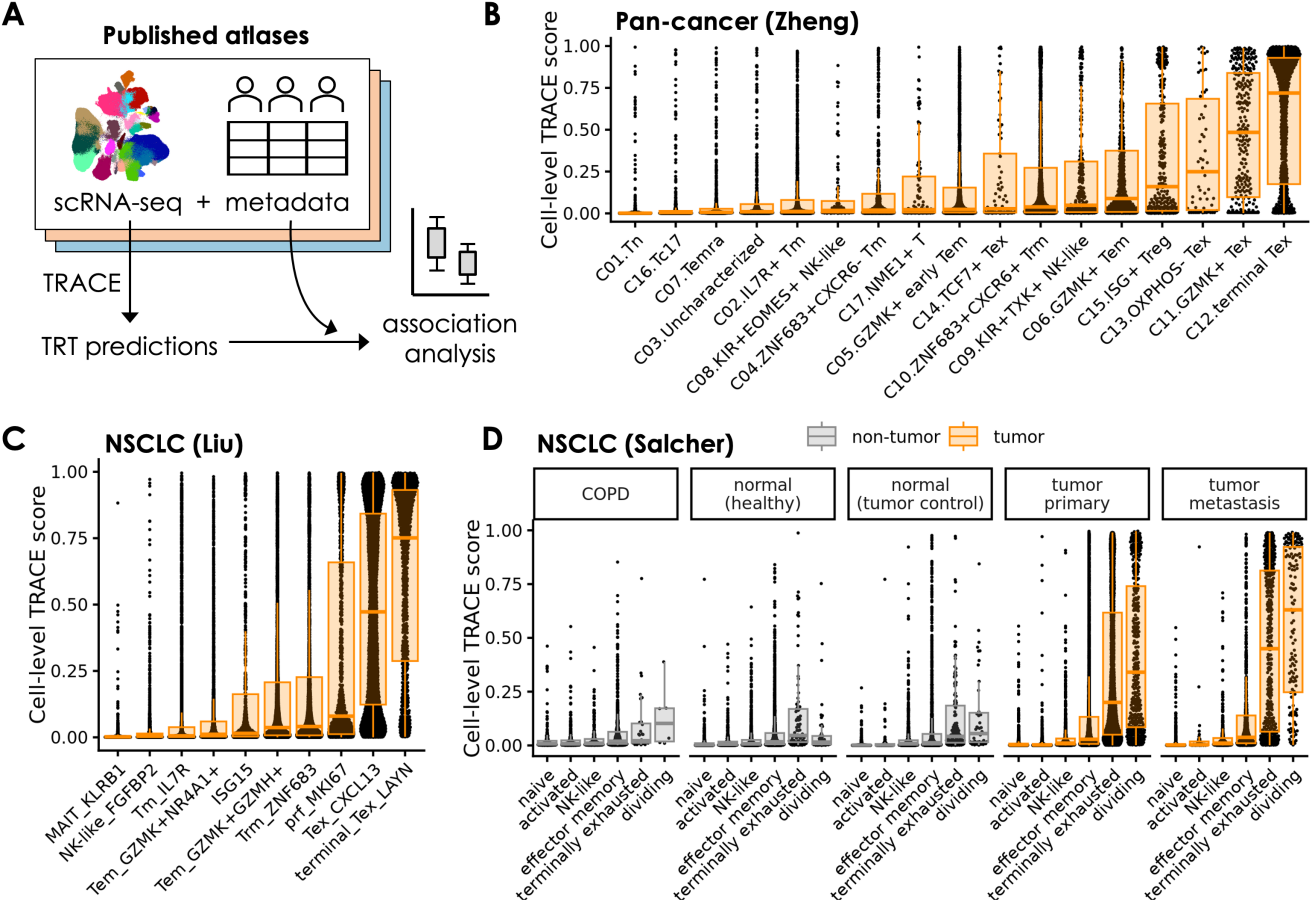
TRACE identifies tumor reactive cells specifically in tumor contexts. **(A)** Application of TRACE to multiple scRNA-seq atlases from the literature. **(B-D)** Distributions of TRACE scores across CD8^+^ cells grouped by CD8^+^ subtype. Tumor cell scores are summarized with orange boxplots, and non-tumor cell scores are summarized with gray boxplots. Cells were subsampled down to 100 cells per sample to ensure relatively even representation of all samples. In B, only CD8^+^ clusters as defined by the original study are included. Scores are shown for the Zheng et al. pan-cancer TIL atlas **(B)**, the Liu et al. NSCLC atlas **(C)**, and the Salcher et al. NSCLC atlas **(D)**.

We used TRACE to evaluate tumor reactive CD8^+^ cells in hundreds of patient samples across multiple indications - including those containing scRNA but no matched scTCR-sequencing data - and interpreted our findings alongside any available tumor or clinical metadata (**Figure 4A**). First, we applied TRACE to individual cells from samples in the Zheng et al. pan-cancer TIL atlas sequenced using the 10x Genomics platform, consisting of 134 patients across 17 cancer types (31). Cells across all patients were pooled together and grouped by CD8^+^ subtype according to the authors’ annotations. As expected, TRACE scores were highest in the terminal, *GZMK*^+^, and OXPHOS^-^ exhausted T cell (T_ex_) subtypes marked by consistent expression of *HAVCR2*, *CTLA4*, and *CXCL13*, but not in *TCF7^+^* T_ex_, suggesting TRACE successfully identifies cells that have undergone chronic antigen stimulation (**Figure 4B**).

TRACE produced a similar pattern when applied to the Liu et al. NSCLC atlas (37) consisting of 234 NSCLC patients treated with neoadjuvant chemo-immunotherapy, with the two exhausted subtypes (T_ex_ *CXCL13* and terminal T_ex_ *LAYN*) showing the highest scores (**Figure 4C**). The two exhausted subtypes, along with the proliferative subtype (prf *MKI67*), showed a bimodal distribution with peaks close to either 1 or 0, consistent with confident TRACE predictions of both tumor reactive and exhausted or dividing bystander cells, respectively. The Zheng et al. and Liu et al. atlases also included some samples with matched scTCR-seq, enabling TRACE scoring at the clone level. In these datasets, expanded clones (>5 cells) were more likely to be predicted as TRT by TRACE than non- or lowly-expanded clones (**Supplementary Figure 4**). Given that TRT clones are associated with antigen-mediated expansion at tumor sites, this provides further support that TRACE captures clones that have undergone activation.

We also applied TRACE to the Salcher et al. NSCLC atlas (38), which contains samples from 151 NSCLC patients with paired normal samples where available, as well as samples from COPD patients and healthy individuals. Similar to previous datasets, CD8^+^ cells from tumor tissue showed the highest TRACE scores in the terminally exhausted and dividing CD8^+^ T cell subtypes (**Figure 4D**). This pattern was remarkably consistent across individual datasets and sequencing platforms within the atlas, including 10x Genomics, BD rhapsody, inDrop, Singleron, and Smart-seq 2 (**Supplementary Figure 5**). Interestingly, CD8^+^ cells from non-tumor tissues showed a different pattern from those in tumor tissue, with relatively few cells predicted to be TRT by TRACE across all CD8^+^subtypes. Thus, TRACE uniquely identified cells in exhausted and dividing transcriptomic states within tumor tissue but not in adjacent normal or inflamed non-tumor contexts. TRACE produced a similar result when applied to the Chu et al. CRC atlas (39), where we observed higher cell-level TRACE scores in *CXCL13*^+^ CD8^+^ cells from CRC samples compared to cells in a similar subtype within normal controls, healthy individuals, or those with inflammatory bowel disease (IBD) (**Supplementary Figure 6**). These observations suggest TRACE precisely identifies cells in tumor reactive states and is not merely a measure of general T cell exhaustion. To determine how well TRACE distinguishes tumor-specific reactive states from non-tumor states in comparison to other tumor reactivity prediction tools, we also scored the Salcher et al. atlas using the gene sets from TRTpred and NeoTCR8 (24, 26). Compared to TRTpred and NeoTCR8, TRACE showed higher separation between exhausted tumor scores and exhausted non-tumor scores, indicating TRACE is highly specific for tumor reactivity relative to other methods (**Supplementary Figure 7**).

### 2.5 TRACE identifies tumor subtypes enriched for TRTs

**Figure 5:**
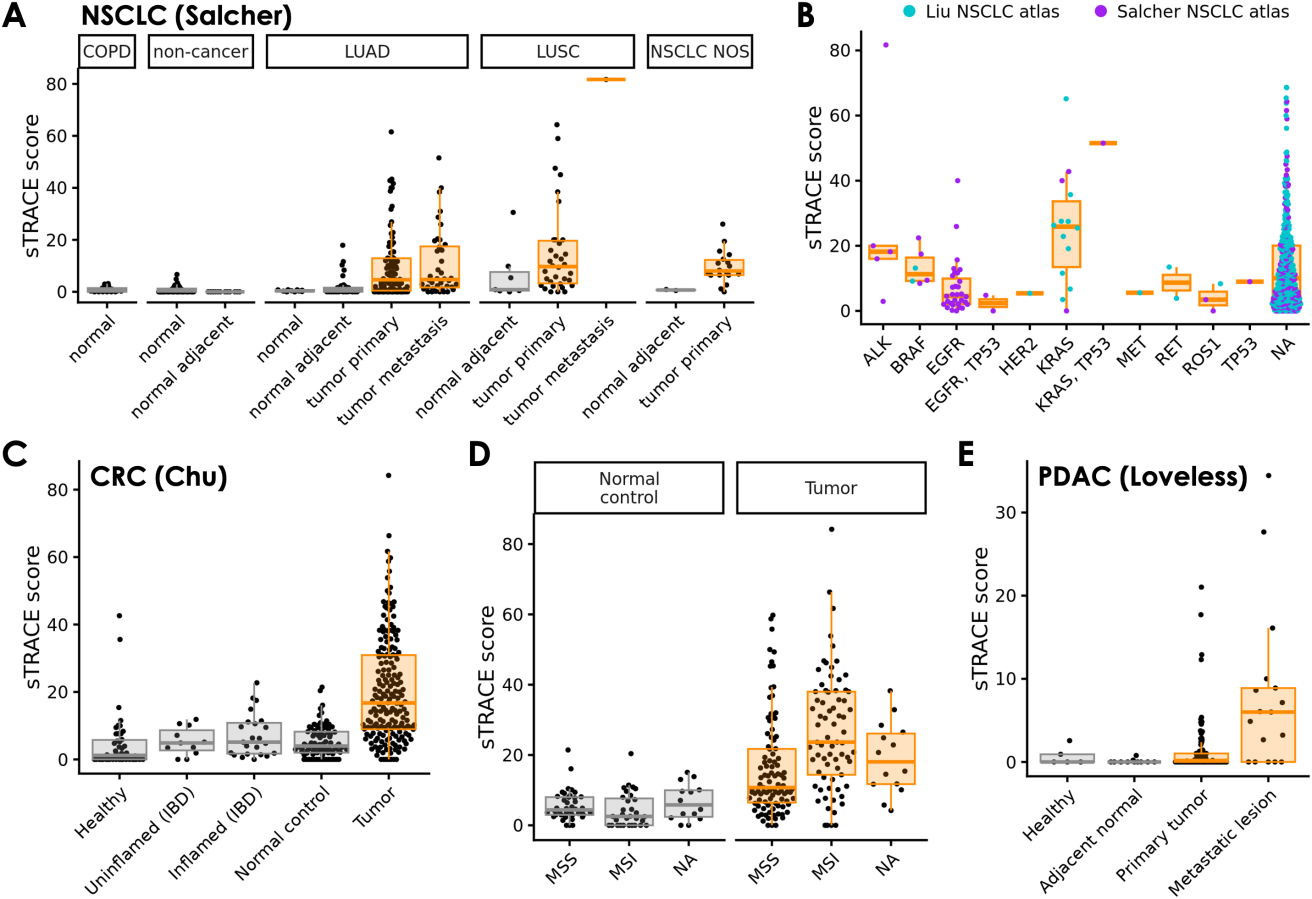
Large-scale survey of tumor reactivity in different cancer types using TRACE. **(A)** sTRACE scores for each NSCLC and non-cancerous sample from the Salcher et al. lung atlas. NOS, not otherwise specified. **(B)** sTRACE scores for NSCLC samples grouped by driver gene status. Samples categorized as NA (not available) were not assessed for one or more of the genes listed. **(C)** sTRACE scores for each CRC and non-cancerous sample from the Chu et al. CRC atlas. **(D)** sTRACE scores for the CRC and normal control samples from panel C grouped by MSI status. Samples categorized as NA (not available) were not assessed for MSI status. **(E)** sTRACE scores for each PDAC and non-cancerous sample from the Loveless et al. PDAC atlas. For all panels, tumor sample scores are summarized with orange boxplots, and non-tumor sample percentages are summarized with gray boxplots.

To compare TRACE scores across samples, we defined a sample-level TRACE score (sTRACE score) as the fraction of CD8^+^ T cells in the sample with a TRACE score of 0.5 or higher. In Salcher et al., tumor samples across NSCLC subtypes (LUAD, LUSC, and unspecified) generally exhibited higher sTRACE scores than non-tumor samples, with false positive sTRACE scores in non-tumor samples of typically < 5% (**Figure 5A**). sTRACE scores were similar between LUAD and LUSC and were also similar between primary and metastatic tumors within LUAD. To assess if certain driver mutations were associated with higher sTRACE scores in NSCLC, we plotted tumor sTRACE scores from Salcher et al. and Liu et al. against listed driver mutations. In both studies, *KRAS* mutations were associated with higher sTRACE scores relative to other reported mutations (**Figure 5B**). High-scoring *KRAS*-mutant samples included both primary and metastatic samples with or without a co-occurring *TP53* mutation.

We similarly applied TRACE to the Chu et al. CRC atlas (39) and found the highest sTRACE scores in tumor samples, with lower scores in normal control and healthy samples and in samples from patients with IBD (**Figure 5C**). Within CRC tumor samples, we further investigated sTRACE scores as a function of MSI status. Colorectal tumors with microsatellite instability (MSI) exhibit higher levels of CD8^+^ T cell infiltration than those that are microsatellite stable (MSS) (40), but the tumor reactivity of these TIL is not well-established. Using TRACE, we found higher sTRACE scores in MSI tumors (p = 5.8x10^-6^; **Figure 5D**), but no differences between normal control samples from MSS or MSI tumors (p = 0.071). Thus, MSI tumors are immunologically favorable in terms of both CD8^+^ T cell infiltration and tumor reactivity. Finally, we scored CD8^+^ cells in the PDAC atlas compiled by Loveless et al. (41). In line with the low immunogenicity of PDAC tumors, we observed lower sTRACE scores overall in these samples. Interestingly, the scores were higher in PDAC metastatic lesions (mostly liver) than primary tumors, despite a decrease in CD8^+^ T cell infiltration in these samples.

## 3 Discussion

Tumor reactive CD8^+^ T cells represent a biologically and clinically meaningful subset of TIL, and their frequency and functionality is a key driver of clinical efficacy of immune checkpoint inhibitors (CPI) and adoptive cell therapies (ACT) in immunologically hot tumors. Identifying and estimating the frequency of TRTs in TIL is of interest as a predictive biomarker of response to CPI or ACT regardless of tissue histology. Further, TRTs can be preferentially isolated for *ex vivo* TIL therapy and for discovery of novel TCR sequences specific to tumor associated antigens.

A growing body of research has shown that TRTs occupy distinct transcriptional states enriched for markers of chronic antigen stimulation, tissue residency, and exhaustion−like programs (e.g., *CXCL13*, *ENTPD1*/CD39, *PDCD1*, *LAG3*, *TOX*). Prior methods - including gene signature scoring methods (e.g., NeoTCR8, MANAscore), marker−based heuristics (e.g., CD39/CD103 co−expression), and machine−learning classifiers trained on individual studies - have advanced the ability to infer tumor reactivity but are often constrained by their dependence on single datasets, lack of model checkpoint availability, or sensitivity to normalization and platform−specific effects. In this study, we introduce TRACE, a clonotype−aware, machine−learning framework designed to infer tumor reactivity from single−cell transcriptomic data while explicitly accounting for technical variability and intra−clonal heterogeneity.

Using autologous co−culture assays and scRNA/scTCR-seq, we confirm that TRACE predictions correspond closely with experimentally reactive clones, providing a strong functional grounding for model performance. TRACE accurately identifies exhausted or chronically stimulated CD8^+^ T−cell populations across NSCLC, CRC, PDAC, and pan−cancer datasets, and distinguishes tumor−specific activation from bystander exhaustion in non−tumor tissues. Associations with clonal expansion and known clinical features (e.g., KRAS mutations, MSI status) underscore biological validity.

By integrating validated TRT and non−TRT clonotypes across multiple TIL and PBMC datasets, TRACE addresses key limitations of previous computational approaches and offers a generalizable strategy for identifying tumor−reactive T cells in both tissue and blood contexts. Inclusion of PBMC clones alongside validated non-TRT TIL and viral-reactive clones in VDJdb increases the diversity of CD8^+^ T-cell phenotypes that constitute the negative label set. This allows the model to sample a larger space of non-TRT phenotypes, including PBMC CD8^+^ T cells that express effector or exhaustion markers in isolation. As an example, an effector gene such as *GZMB* can be expressed in both TRT TILs and PBMCs, and including PBMCs in the negative set allows the appropriate weight to be applied to that feature. Thus, TRACE is suitable for application to a variety of contexts beyond primary tumor samples, such as TRT scoring of cells derived from lymph nodes or normal adjacent tissues. Since TRACE is trained on a relatively small set of validated TRT clones even after aggregation of multiple datasets, there is potential for this model to be refined on new datasets such as rare cancer subtypes, or on tumors with atypical TIL transcriptional states following multiple rounds of treatment. Openly sharing the code, model weights, and feature set allows anyone in the community to develop model applications on public atlases or niche datasets. Further, there is potential for this model to be refined with additional autologous co-culture experiments from additional donors across multiple indications, with emphasis on identifying tumor reactive clonotypes with atypical profiles.

We envision multiple applications of clonal and sample TRACE scores to study the heterogeneity of TRTs within and between tumor samples. Clone-resolved TRACE scores could be integrated with complementary clonal metadata, including experimentally determined or inferred antigen specificity, transcriptional T cell states derived from scRNA-seq, and epigenetic programs inferred from scATAC-seq. Such analyses may enable finer dissection of functional heterogeneity among tumor-reactive clonotypes, distinguishing, for example, exhausted, proliferative, stem-like, or tissue-resident programs within antigen-experienced T cell populations. In addition, integrating clonal TRACE scores with spatial transcriptomics or imaging-based assays could provide insight into the spatial organization of tumor-reactive T cells within the tumor microenvironment, including their proximity to tumor cells, stromal niches, or immunosuppressive myeloid populations. This may further enable investigation of cell–cell interactions and signaling networks that shape T cell dysfunction or persistence *in situ*. Identifying shared TCR clonotypes enriched among highly TRACE^+^ tumor-reactive TILs could facilitate discovery and prioritization of candidates for adoptive cell therapies, including TCR-T and TIL products.

Summarizing clonal or single-cell TRACE scores to sample level sTRACE scores enables rapid interrogation of scRNA-seq TIL atlases across multiple indications. Integrating sTRACE scores with sample-level clinical metadata can provide insight into association between tumor-reactive TILs and response to immune checkpoint blockade, either as a predictive biomarker hypothesis or to characterize tumor microenvironment (TME) states. Association with TME subtypes, somatic alterations (e.g. KRAS or EGFR mutations in lung cancer), genomic features (e.g. MSI vs MSS in colorectal cancer) or proxies of neo-antigen burden such as tumor mutational burden (TMB) may further contextualize the therapeutic response, or lack thereof, of these tumors to immunomodulatory therapies. High-TRT tumors stratified by histology, treatment status or TME, once validated in independent cohorts, could inform clinical trial enrollment or indication expansion for TIL therapies. In addition, identifying TRT clonotypes from tumor biopsies and tracking them in blood or tumor tissue following immunotherapy may enable the investigation of TRT clonal breadth and depth and their relationship with response quality.

In conclusion, TRACE is a framework that will be of general interest to the adoptive cell therapy field.

## 4 Materials and methods

### 4.1 Data aggregation and cleaning

For all datasets used in this study except two (34, 35), we downloaded raw sequencing reads (FASTQ format) to partially mitigate batch effects by ensuring consistent alignment and processing of the reads. When starting with FASTQ files, 10x Genomics Cell Ranger v8.0.1 was used for gene expression quantification, TCR sequence assembly, and extraction of cell barcodes and UMIs. RNA was aligned to a modified version of the GRCh38 reference containing only protein-coding genes, and TCR sequences were aligned to the vdj_GRCh38_alts_ensembl reference. For all datasets, Seurat (version 5.1.0) (42) was used to process feature-barcode matrices, align gene expression and TCR datasets, filter cells, identify and annotate clusters, and perform differential gene expression analyses. Briefly, cells containing fewer than 200 unique genes and cells where the fraction of counts belonging to mitochondrial genes (%mito) exceeded 25% were removed. In addition, cells were scored for how well they expressed S- and G2M-phase gene sets using CellCycleScoring (Seurat). For each cell, a value CCdifference was calculated as the difference between that cell’s S-phase and G2M-phase scores. scRepertoire (43) was used to ensure a maximum of one TCRα and one TCRβ chain per cell.

Where possible, we used experimentally verified TRT and non-TRT labels identified using paired TCRαβ CDR3 sequences provided by the authors. When only TRT TCRβ CDR3 sequences were provided (22), tumor reactivity labels were assigned based on CDR3β sequences alone. For some datasets, we labeled additional clones as likely non-tumor reactive if they contained a CDR3β chain with an annotated target in VDJdb.

After removing clones with unknown TRT status, we further filtered out 1) putative CD4^+^ T cells and 2) samples for which the number of annotated clones was low. CD4^+^ cells were identified based on the relative expressions of *CD4* and *CD8A* after clustering. To cluster the cells, we followed the scTransform workflow (Seurat), regressing %mito and CCdifference. TCR variable and joining genes were removed from the list of genes used for principal component analysis and clustering to avoid clonotype-specific clusters. Dimensionality reduction was performed with 20 principal components, and clustering was performed with resolutions typically between 0.5 and 1.0.

### 4.2 Data filtering

The assembled dataset contains expression data from 30,560 genes and 40,156 cells representing 16,465 distinct CD8^+^ clones. Prior to model training, the following gene filtering steps were applied to the dataset – 1) selection of genes that are expressed (> 0 count) in at least 10% of cells across the dataset, 2) selection of top 5,000 most highly variable genes using FindVariableGenes (Seurat) and 3) exclusion of mitochondrial, ncRNA, ribosomal and other highly expressed genes (**Supplementary Methods**). 966 genes meeting these criteria were retained for model training. No cells were excluded and the clone labels (TRT vs non-TRT) were not used at any point during filtering.

### 4.3 Clone-level feature construction

Single-cell expression matrices were summarized to clone-level feature vectors by aggregating expression across all cells assigned to the same clone. Clone expression was summarized using a percentile-based aggregation (75^th^ percentile) to provide a robust clone-level representation while reducing sensitivity to outlier cells. Genes used for model development were drawn from a predefined feature universe defined in the data filtering step. The parameter (75^th^ percentile) for clone collapsing was selected after assessing the results of optimization experiment 1 as described in supplementary methods, where several percentiles were evaluated. In addition, raw counts for genes in each cell were transformed either by expression binning (eb) or log normalization (ln). Expression binning involves binning genes by their non-zero expression count in each cell into *n* equally sized bins (default 20). All genes with zero counts were grouped into the 0^th^ bin, and each gene’s expression value was represented by their bin count from 0 to n. Expression binning elegantly deals with variation in counts data due to variability in sample batching, library preparations, read depth, or sequencing platforms which frequently plagues single-cell RNAs data analysis. Log normalization involves a transformation of gene expression values within each cell using $\log_{2}(10000*\frac{count}{sum(counts)}+1)$. Importantly, neither method requires cell or data set normalization or transformation, such as scTransform. Expression binning was chosen as the default method for pre-processing after clone-level summarization, as it was superior to log normalization (optimization experiment 1) across a range of hyperparameters and resulted in > 5X faster training compared to log normalization.

### 4.4 Model class and training objective

We used an XGBoost gradient-boosted decision tree classifier. XGBoost was chosen as the top performing model in a head-to-head comparison with randomForest and AdaBoost (**Supplementary Methods**). Because tumor reactive clones represent the clinically relevant positive class and were present at significantly lower prevalence than negative class in the dataset, model selection emphasized positive-class performance, and evaluation focused on positive-class–aware metrics (including PR-AUC and F1 for the positive class), in addition to overall accuracy.

### 4.5 Cross-validation design and prevention of leakage

To obtain an unbiased estimate of generalization and prevent information leakage from feature selection and hyperparameter tuning, we used nested cross-validation at the clone level. In each outer fold, 20% of the entire dataset was held out for model validation and was not used for model training. 80% of the dataset was used as training data for inner cross-validation. 5-fold cross-validation was performed on the inner fold training data for hyperparameter optimization and feature selection, and the optimal model was re-trained on the full 80% training data and tested on the 20% hold-out data for reporting model performance. The outer fold (training-test-validation) was repeated between 3–50 times for different optimization experiments. During each inner fold cross-validation, TRT positive class and TRT negative class clones were balanced at 10:90 ratio by subsampling negative class clones.

### 4.6 Hyperparameter optimization

Hyperparameters for XGBoost, randomForest and AdaBoost were optimized within the inner loop using automated search. The TRACE framework supports both randomized search and Bayesian optimization (Optuna); hyperparameter optimization was performed and fold-specific optimal configurations were consolidated using a stability-based selection strategy to reduce overfitting to any single split. The “Optuna” method is default as it had superior performance compared to “random grid search” (**Supplementary Methods**) although both options are available when running TRACE.

### 4.7 Feature selection and “freezing” of the signature

To improve interpretability and reduce overfitting, we performed cross-validated feature selection using model-derived feature importance computed within the training folds. Importance values were converted to per-fold ranks and aggregated across folds, and a fixed set of genes was selected and then frozen for final model fitting on inner training data and downstream evaluation on hold out validation data. The number of genes to be selected was chosen to be 50 (**Supplementary Methods**). These selected features, in addition to the additional 450 features used to establish the bins - are stored with the trained model and used for consistent feature alignment during inference on new datasets.

### 4.8 Performance reporting

Model performance was summarized across outer folds (mean ± standard deviation, unless otherwise indicated) and reported on the held-out validation set. Metrics included threshold-independent discrimination (ROC-AUC, PR-AUC) and threshold-dependent classification performance, with particular emphasis on positive-class precision/recall tradeoffs relevant to tumor reactive clone identification.

### 4.9 Benchmarking

We implemented other published TRT methods from datasets used in this study (NeoTCR8, TRTpred, TR30, and MANAscore) and applied them to our extended dataset.

For the NeoTCR8, TRTpred, and TR30 methods, we 1) scored individual cells in our dataset using their published gene lists and chosen scoring method, 2) calculated clone-level scores from these cell-level scores, 3) trained a logistic regression model using training set clones in each outer fold, and 4) applied these models to predict TRT labels for held-out clones, using the threshold that best separated TRT from non-TRT clones within the training set.

For NeoTCR8, individual cell scores were found using single sample GSEA. To apply NeoTCR8 to multiple training sets without clustering, the clone-wise score was set to be the maximum score found within that clone. Within each seed, the predicted clone labels were found by comparing test set scores to the optimal threshold learned from the training set, maximizing Youden’s J.

For TRTpred, individual cell scores were found using Singscore, as no single method was clearly described. The clone-wise TRTpred score was set to be the maximum score found within that clone. Within each seed, scores were standardized based on the distribution of scores in the training set (mean ± SD), and the same transformation was applied to scores from the test set. The predicted clone labels were identified by comparing the standardized test set scores to the optimal threshold learned from the training set, maximizing accuracy.

For TR30, individual cell scores were found using the UCell package. The clone-wise TR30 score was set to be the mean score found within that clone. Within each seed, the predicted clone labels were identified by comparing test set scores to the optimal threshold learned from the training set, maximizing Youden’s J.

For MANAscore, we used code available in their Github repository to find imputed and non-imputed MANAscores for each cell and called cells scoring higher than the level of the final trough for each score as MANAscore_hi_ cells. Putative tumor reactive clones were defined as those with at least 5 MANAscore_hi_ cells. For a few samples, the provided code was unable to identify the final imputed MANAscore trough; for these samples, we set these thresholds equal to the value of the matched non-imputed MANAscore trough. To compare clone-level TRT scores between methods, we set the clone-level score equal to the maximum imputed MANAscore found within a clone.

We validated TRACE using the Tan et al. BT21 dataset, which contained 5’ scRNA-seq but where scTCR-seq was not publicly available. We first assigned clone labels using the TRAV and TRBV gene with the most counts detected within each cell. In cases of ties, we selected the first pair present in the list. This yielded 33 clones (TRAV-TRBV pairs) matching those in the list of 46 TRT clones published by the authors.

### 4.10 TRACE scoring of public scRNA-seq atlases

Counts were downloaded for each dataset and converted to h5ad where necessary. CD8^+^ cells were selected using the cell type annotations provided by the study authors except in the case of the Loveless et al. PDAC atlas, for which CD8^+^ labels were unavailable and were therefore assigned by identifying clusters high for *CD8A/CD8B* expression. TRACE was run in single-cell mode using the config_preprocess_sc.yaml settings with the gene_selection_method set to “all”. For the Zheng et al. pan-cancer and Liu et al. NSCLC atlases, TRACE was additionally run at the clone level after integrating the scTCR-seq data. TRACE was applied to the raw counts for each atlas, except for the Chu et al. CRC atlas, which only contained normalized counts. Resulting TRACE scores were then joined with the patient metadata available from each study. For sample-level sTRACE scores, only samples with ≥ 10 CD8^+^ cells were considered. The Becker et al. dataset was removed from the Chu et al. CRC atlas since sequencing was performed on single nuclei rather than single cells (44). Additional details on any pre-processing of the atlases can be found in the **Supplementary Methods**, and the code for score generation is available at https://github.com/ksqtx/trace.

### 4.11 TIL/tumor autologous co-culture assay

Melanoma tumor fragments were digested and used to develop a melanoma tumor cell line. TIL from tumor fragments were expanded *ex vivo* following protocols described elsewhere and edited for SOCS1 and/or Regnase-1 or left unedited (NoEP) before cryopreservation (45, 46). After thaw, expanded TIL were rested for 24 h prior to co-culture with the matched autologous melanoma cell line at an E:T ratio of 0.5:1 (300,000 T cells and 600,000 tumor cells per well) for 20 h. To evaluate MHC-mediated T cell activation, an MHC-block control condition was used, which involved incubating tumor cells with anti-MHC-I (168 ug/mL, Ultra-LEAF clone W6/32, BioLegend) and anti-MHC-II (50 ug/mL, BD Pharmingen clone Tu39, BD) at 37 °C for 1 h prior to co-culture.

Prior to sequencing, T cells from tumor starting material and from autologous co-cultures underwent dead cell removal (#130-090-101, Miltenyi Biotec) followed by T cell enrichment using CD3 microbeads (EasySep Human CD3 #17851, STEMCELL Technologies). Sequencing was performed using the 10x Genomics platform and RNA and VDJ libraries were prepared by the Chromium Next GEM Single Cell 5’v2 HT Reagent kit according to the manufacturer’s protocol (10x Genomics).

We performed scRNA/scTCR-seq on 4 co-culture samples (one from each of 4 edit-groups) and one +MHC block sample. We used the +MHC-block condition to identify a unique *TNFRSF9*-high transcriptional state enriched in the unblocked co-culture samples. Expt. TRT clones were identified as any clone with at least 1 cell and 5% of cells in a *TNFRSF9*-high transcriptional state in any replicate.

## Data Availability

The datasets presented in this study can be found in online repositories. The names of the repository/repositories and accession number(s) can be found in the article/**Supplementary Material**. Feature-barcode matrices and clone ID’s for the tumor sample used in the autologous co-culture experiment have been deposited at Zenodo (DOI: 10.5281/zenodo.19096905). Requests for additional data can be sent to datasharingrequest@ksqtx.com. The TRACE model, model checkpoint weights, and instructions for its use can be found at https://github.com/ksqtx/trace. TRACE scores for the atlases shown in **Figures 4** and **5** are available at https://github.com/ksqtx/trace.
